# Association Between Serious Hypoglycemia and Calcium-Channel Blockers Used Concomitantly With Insulin Secretagogues

**DOI:** 10.1001/jamanetworkopen.2021.24443

**Published:** 2021-09-02

**Authors:** Young Hee Nam, Colleen M. Brensinger, Warren B. Bilker, James H. Flory, Charles E. Leonard, Sean Hennessy

**Affiliations:** 1 Therapeutics Research and Infectious Disease Epidemiology (TIDE), Department of Population Medicine, Harvard Medical School and Harvard Pilgrim Health Care Institute, Boston, Massachusetts; 2 Center for Pharmacoepidemiology Research and Training, Center for Clinical Epidemiology and Biostatistics, Department of Biostatistics, Epidemiology and Informatics, Perelman School of Medicine, University of Pennsylvania, Philadelphia; 3 Endocrinology Service, Department of Subspecialty Medicine, Memorial Sloan Kettering Cancer Center, New York, New York

## Abstract

This cohort study investigates whether serious hypoglycemia is reduced in a Medicaid population receiving calcium-channel blockers with insulin secretagogues.

## Introduction

Serious hypoglycemia is a major, potentially fatal adverse event caused by insulin secretagogues.^1^ Previous case reports suggested that calcium-channel blockers (CCBs) might reduce the risk of serious hypoglycemia in patients with hyperinsulinemic hypoglycemia.^2,3^ However, the association of serious hypoglycemia and CCBs used with insulin secretagogues has remained unclear. Because insulin secretion by the pancreas is mediated by calcium influx in beta cells through calcium channels,^4^ we conducted a population-based observational study on the hypothesis that concomitant use of CCBs may be associated with reduced rates of serious hypoglycemia in insulin secretagogue users.

## Methods

This self-controlled case series study was approved by the institutional review board of the University of Pennsylvania, which waived the requirement for informed consent because the use or disclosure of the protected health information involved no more than minimal risk to the privacy of individuals, and the research could not practicably be conducted without the waiver or alteration and without access to and use of the protected health information. We followed the Strengthening the Reporting of Observational Studies in Epidemiology (STROBE) reporting guideline. We used claims data from the Medicaid programs of 5 US states (California, Florida, New York, Ohio, and Pennsylvania, encompassing more than a third of the nationwide Medicaid population), supplemented with Medicare claims for dual enrollees, from January 1, 1999, to December 31, 2011, and used the self-controlled case series design. Our methods were described in detail in a previous study.^5^ Users (aged ≥18 years) of insulin secretagogues (glimepiride, glipizide, glyburide, nateglinide, and repaglinide; object drugs) or metformin (negative-control object drug) who were enrolled in Medicaid for at least 180 days immediately before the first object drug–specific observation time (eFigure 1 in the Supplement), who were free of an enrollment gap and dispensing of that object drug, and who experienced at least 1 outcome event during the observation time were eligible (flowchart in eFigure 2 in the Supplement). The exposure was active prescription for a CCB (amlodipine, diltiazem, felodipine, isradipine, nicardipine, nifedipine, nimodipine, nisoldipine, and verapamil; precipitant drugs) during the observation time. The outcome was serious hypoglycemia ascertained by *International Classification of Diseases, Ninth Revision, Clinical Modification* discharge diagnosis codes appearing in principal position on inpatient hospital claims or any position on emergency department claims (positive predictive value of approximately 78%-89%).^5^ The self-controlled case series design inherently controls for all time-invariant confounders, and we further controlled for time-varying potential confounders (eTables 1 and 2 in the Supplement) at the person-day level. We computed confounder-adjusted rate ratios (RRs) and 95% CIs for outcome occurrence during precipitant-exposed vs -unexposed time using conditional Poisson regression, and we performed sensitivity analyses. Statistical analysis was conducted from April 8, 2018, to August 30, 2018.

## Results

The number of individuals who met inclusion criteria and the patient characteristics for each object drug–specific sample are presented in Table 1. The median age at the start of observation ranged from 63.2 years (interquartile range [IQR], 50.0-73.8 years) for metformin to 72.8 years (IQR, 63.2-80.8 years) for repaglinide, and the proportion of women and men ranged from 7100 women (63.8%) and 4036 men (36.2%) for glipizide to 4362 women (67.7%) and 2080 men (32.3%) for glimepiride. Adjusted RRs (95% CIs) for serious hypoglycemia with (vs without) concomitant CCB use were reduced with glimepiride (0.79; 0.70-0.89), glipizide (0.86; 0.79-0.94), glyburide (0.81; 0.73-0.90), and metformin (0.91; 0.86-0.96). Adjusted RRs (95% CIs) with nateglinide and repaglinide were 0.74 (0.55-1.00) and 0.84 (0.69-1.02), respectively (Table 2). Adjusted RRs (95% CIs) for sensitivity analyses 1 and 2, respectively, were similar: glimepiride 0.79 (0.71-0.89) and 0.82 (0.70-0.95), glipizide 0.86 (0.79-0.94) and 0.80 (0.71-0.90), glyburide 0.81 (0.73-0.89) and 0.81 (0.71-0.92), nateglinide 0.69 (0.52-0.93) and 0.76 (0.52-1.09), repaglinide 0.89 (0.73-1.07) and 0.78 (0.61-0.98), and metformin 0.91 (0.86-0.96) and 0.91 (0.84-0.99) (Table 2).

**Table 1.** Characteristics of the Study Sample by Object Drug

Characteristic	No. (%)					
	Glimepiride	Glipizide	Glyburide	Nateglinide	Repaglinide	Metformin
Persons, No.	6442	11 136	8865	1112	2085	24 594
Person-days of observation time						
Per individual, median (IQR)	128.0 (60.0-290.0)	127.0 (60.0-289.0)	104.0 (42.0-234.0)	92.0 (38.0-205.0)	93.0 (38.0-196.0)	206.0 (97.0-440.0)
Total	1 524 588	2 604 469	1 799 555	203 173	359 615	8 707 447
Outcome occurrences during observation time, No.	8159	14 353	11 129	1595	2901	32 639
Persons throughout the observation time						
Precipitant exposed	1075 (16.7)	1676 (15.1)	1388 (15.7)	228 (20.5)	382 (18.3)	2241 (9.1)
Precipitant unexposed	3449 (53.5)	6038 (54.2)	5026 (56.7)	598 (53.8)	1097 (52.6)	15 397 (62.6)
Demographic characteristics						
Age at start of observation time, median (IQR), y	71.4 (60.8-79.8)	68.4 (56.6-78.1)	69.7 (58.1-79.2)	70.5 (59.7-79.1)	72.8 (63.2-80.8)	63.2 (50.0-73.8)
Sex						
Men	2080 (32.3)	4036 (36.2)	3197 (36.1)	381 (34.3)	681 (32.7)	8703 (35.4)
Women	4362 (67.7)	7100 (63.8)	5668 (63.9)	731 (65.7)	1404 (67.3)	15 891 (64.6)
State of residence						
California	3176 (49.3)	5318 (47.8)	5060 (57.1)	561 (50.4)	828 (39.7)	12 181 (49.5)
Florida	660 (10.2)	1320 (11.9)	727 (8.2)	112 (10.1)	207 (9.9)	2294 (9.3)
New York	1248 (19.4)	2578 (23.2)	1696 (19.1)	226 (20.3)	690 (33.1)	5677 (23.1)
Ohio	949 (14.7)	1155 (10.4)	956 (10.8)	148 (13.3)	121 (5.8)	2792 (11.4)
Pennsylvania	409 (6.3)	765 (6.9)	426 (4.8)	65 (5.8)	239 (11.5)	1650 (6.7)
Calendar year at start of observation time						
1999	169 (2.6)	436 (3.9)	494 (5.6)	0	91 (4.4)	1213 (4.9)
2000	351 (5.4)	946 (8.5)	857 (9.7)	0	133 (6.4)	2406 (9.8)
2001	371 (5.8)	902 (8.1)	748 (8.4)	87 (7.8)	169 (8.1)	2408 (9.8)
2002	457 (7.1)	910 (8.2)	747 (8.4)	110 (9.9)	165 (7.9)	2011 (8.2)
2003	581 (9.0)	908 (8.2)	789 (8.9)	124 (11.2)	170 (8.2)	2012 (8.2)
2004	564 (8.8)	899 (8.1)	795 (9.0)	131 (11.8)	151 (7.2)	1994 (8.1)
2005	575 (8.9)	993 (8.9)	783 (8.8)	180 (16.2)	147 (7.1)	2065 (8.4)
2006	784 (12.2)	1260 (11.3)	956 (10.8)	122 (11.0)	243 (11.7)	2605 (10.6)
2007	668 (10.4)	936 (8.4)	693 (7.8)	102 (9.2)	215 (10.3)	1826 (7.4)
2008	520 (8.1)	769 (6.9)	572 (6.5)	85 (7.6)	205 (9.8)	1651 (6.7)
2009	534 (8.3)	907 (8.1)	542 (6.1)	74 (6.7)	142 (6.8)	1874 (7.6)
2010	516 (8.0)	755 (6.8)	538 (6.1)	56 (5.0)	145 (7.0)	1530 (6.2)
2011	352 (5.5)	515 (4.6)	351 (4.0)	41 (3.7)	109 (5.2)	999 (4.1)
Medicare dual enrollment at start of observation time	5165 (80.2)	8202 (73.7)	6531 (73.7)	908 (81.7)	1763 (84.6)	15 839 (64.4)
Nursing home resident at start of observation time	801 (12.4)	1648 (14.8)	1148 (12.9)	202 (18.2)	318 (15.3)	2261 (9.2)
Exposure to precipitant drugs, person-days						
Any CCB	516 301 (33.9)	881 870 (33.9)	600 507 (33.4)	76 525 (37.7)	126 606 (35.2)	2 254 403 (25.9)
Amlodipine	331 629 (21.8)	530 115 (20.4)	345 737 (19.2)	50 848 (25.0)	83 193 (23.1)	1 352 202 (15.5)
Diltiazem	81 366 (5.3)	127 966 (4.9)	101 961 (5.7)	9286 (4.6)	18 798 (5.2)	364 120 (4.2)
Felodipine	10 874 (0.7)	30 600 (1.2)	12 555 (0.7)	482 (0.2)	1340 (0.4)	53 097 (0.6)
Isradipine	2633 (0.2)	1424 (0.1)	1597 (0.1)	140 (0.1)	623 (0.2)	8922 (0.1)
Nicardipine	953 (0.1)	229 (0.009)	109 (0.006)	67 (0.03)	0	2009 (0.02)
Nifedipine	58 281 (3.8)	150 030 (5.8)	100 528 (5.6)	11 766 (5.8)	16 500 (4.6)	297 457 (3.4)
Nimodipine	0	0	24 (0.001)	0	0	10 (0.0001)
Nisoldipine	5294 (0.3)	5011 (0.2)	3969 (0.2)	209 (0.1)	686 (0.2)	21 639 (0.2)
Verapamil	25 271 (1.7)	36 495 (1.4)	34 027 (1.9)	3727 (1.8)	5466 (1.5)	154 947 (1.8)
Time-varying covariates^a^						
Acute infection in prior 15 d	210 315 (13.8)	372 181 (14.3)	242 328 (13.5)	35 813 (17.6)	59 448 (16.5)	959 180 (11.0)
Atypical antipsychotics in prior 30 d	156 683 (10.3)	336 643 (12.9)	210 480 (11.7)	32 602 (16.0)	35 983 (10.0)	1 655 554 (19.0)
Calcineurin inhibitors in prior 30 d	4738 (0.3)	16 929 (0.6)	7770 (0.4)	2381 (1.2)	1513 (0.4)	10 735 (0.1)
Corticosteroids in prior 30 d	91 295 (6.0)	151 595 (5.8)	106 712 (5.9)	15 557 (7.7)	25 074 (7.0)	399 114 (4.6)
CYP2C9 inhibitors in prior 30 d	192 273 (12.6)	322 629 (12.4)	201 423 (11.2)	32 490 (16.0)	43 151 (12.0)	1 034 732 (11.9)
CYP3A4 inhibitors in prior 30 d	288 421 (18.9)	474 634 (18.2)	276 337 (15.4)	39 587 (19.5)	80 354 (22.3)	1 726 004 (19.8)
Furosemide in prior 30 d	421 501 (27.6)	681 447 (26.2)	428 724 (23.8)	59 318 (29.2)	97 680 (27.2)	1 399 156 (16.1)
Insulin in prior 30 d	362 443 (23.8)	608 649 (23.4)	304 662 (16.9)	83 309 (41.0)	137 844 (38.3)	2 323 606 (26.7)
Noninsulin other antidiabetics in prior 30 d	391 065 (25.7)	449 326 (17.3)	260 728 (14.5)	56 785 (27.9)	74 295 (20.7)	1 924 117 (22.1)
Other drugs that can cause hypoglycemia in prior 30 d	31 284 (2.1)	79 789 (3.1)	51 982 (2.9)	5572 (2.7)	7190 (2.0)	297 895 (3.4)
Other anti-infectives in prior 15 d	136 410 (8.9)	242 749 (9.3)	157 311 (8.7)	21 522 (10.6)	31 760 (8.8)	746 558 (8.6)
Protease inhibitors in prior 30 d	2404 (0.2)	12 988 (0.5)	7995 (0.4)	1312 (0.6)	1171 (0.3)	53 009 (0.6)
Quinolones in prior 15 d	59 887 (3.9)	93 014 (3.6)	63 653 (3.5)	9499 (4.7)	15 689 (4.4)	241 389 (2.8)
Salicylates in prior 30 d	191 960 (12.6)	316 864 (12.2)	210 910 (11.7)	33 891 (16.7)	45 884 (12.8)	1 185 306 (13.6)
Thiazide diuretics in prior 30 d	223 688 (14.7)	343 242 (13.2)	227 188 (12.6)	28 332 (13.9)	53 142 (14.8)	1 390 700 (16.0)

Abbreviations: CCB, calcium-channel blocker; CYP, cytochrome P450 enzyme; IQR, interquartile range.

^a^ Exposure to prespecified time-varying covariates: assessed at the person-day level during the observation time. Details on each time-varying covariate are presented in eTable 1 in the Supplement.

**Table 2.** Adjusted Rate Ratios for Serious Hypoglycemia With vs Without Use of Calcium-Channel Blockers in Insulin Secretagogue or Metformin Users

Object drug	During observation time		During precipitant-exposed time^a^		Rate ratio (95% CI)^b^
	Person-days	Outcomes	Person-days	Outcomes	
Primary analyses					
Glimepiride	1 524 588	8159	516 301	2847	0.79 (0.70-0.89)
Glipizide	2 604 469	14 353	881 870	4844	0.86 (0.79-0.94)
Glyburide	1 799 555	11 129	600 507	3627	0.81 (0.73-0.90)
Nateglinide	203 173	1595	76 525	607	0.74 (0.55-1.00)
Repaglinide	359 615	2901	126 606	1044	0.84 (0.69-1.02)
Metformin	8 707 447	32 639	2 254 403	8220	0.91 (0.86-0.96)
Sensitivity analysis 1^c^					
Glimepiride	1 597 829	8633	540 472	3005	0.79 (0.71-0.89)
Glipizide	2 748 382	15 383	932 634	5176	0.86 (0.79-0.94)
Glyburide	1 892 422	11 878	624 668	3829	0.81 (0.73-0.89)
Nateglinide	213 845	1677	80 219	634	0.69 (0.52-0.93)
Repaglinide	380 387	3062	134 359	1119	0.89 (0.73-1.07)
Metformin	8 970 714	33 791	2 326 204	8536	0.91 (0.86-0.96)
Sensitivity analysis 2^d^					
Glimepiride	926 395	5187	309 876	1822	0.82 (0.70-0.95)
Glipizide	1 385 627	8236	479 428	2815	0.80 (0.71-0.90)
Glyburide	1 076 918	6991	366 325	2328	0.81 (0.71-0.92)
Nateglinide	145 698	1193	52 613	447	0.76 (0.52-1.09)
Repaglinide	224 581	1950	77 036	681	0.78 (0.61-0.98)
Metformin	4 424 943	17 143	1 187 805	4544	0.91 (0.84-0.99)

^a^ Precipitant-exposed time: days of concomitant use with a precipitant drug (ie, calcium-channel blockers) during observation time.

^b^ Rate ratio: [(outcome occurrence rate during precipitant-exposed time)/(outcome occurrence rate during precipitant-unexposed time)]; adjusted for potential confounders (eTable 1 in the Supplement).

^c^ Sensitivity analysis 1 additionally included observation time during which death occurred.

^d^ Sensitivity analysis 2 excluded observation time during which death occurred and observation time with potentially incomplete data (defined operationally as observation time during which a person had a managed care plan, a private health insurance, or restricted benefits, and among Medicaid-Medicare dual enrollees, during which a person was enrolled in a group health organization or a Medicare Advantage plan for which the Centers for Medicare and Medicaid Services does not process provider claims).

## Discussion

Calcium-channel blockers used concomitantly with insulin secretagogues were associated with reduced rates of serious hypoglycemia compared with the use of insulin secretagogues without CCBs. Although the reduced RRs for metformin suggest an inherent effect of CCBs rather than a drug interaction, further studies are needed to elucidate the mechanism. Limitations of this study include lack of data on actual intake of drugs, lifestyle, and health behaviors; however, such factors would have introduced bias only if they were varying within persons across the CCB-exposed and -unexposed time and associated with both CCB use and serious hypoglycemia. Generalizability beyond the Medicaid population needs further investigation. Our findings may help clinicians with cardiovascular drug choice for patients treated with insulin secretagogues. Our findings also suggest that it may be important to monitor whether glycemic control is sufficient without dose adjustments when patients receiving glucose-lowering agents are coadministered a CCB.

## Notes

Supplement. **eFigure 1.** Observation time **eFigure 2.** Flowchart of the identification of study samples **eTable 1.** Prespecified time-varying potential confounders controlled for with conditional Poisson regression model **eTable 2.***ICD-9-CM* diagnosis codes used to identify acute infections
