# Supplementary material for: Association Between Serious Hypoglycemia and Calcium-Channel Blockers Used Concomitantly With Insulin Secretagogues
Source: JAMA Netw Open. 2021 Sep 2;4(9):e2124443. doi: 10.1001/jamanetworkopen.2021.24443 (PMC8414188; doi:10.1001/jamanetworkopen.2021.24443)
Supplement: Supplement. — eFigure 1. Observation time eFigure 2. Flowchart of the identification of study samples eTable 1. Prespecified time-varying potential confounders controlled for with conditional Poisson regression model eTable 2.ICD-9-CM diagnosis codes used to identify acute infections [file jamanetwopen-e2124443-s001.pdf]

Supplemental Online Content

Nam YH, Brensinger CM, Bilker WB, Flory JH, Leonard CE, Hennessy S. Association between serious hypoglycemia and calcium-channel blockers used concomitantly with insulin secretagogues. *JAMA Netw Open*. 2021;4(9):e2124443.
doi:10.1001/jamanetworkopen.2021.24443

eFigure 1. Observation time

eFigure 2. Flowchart of the identification of study samples

eTable 1. Prespecified time-varying potential confounders controlled for with conditional Poisson regression model

eTable 2. *ICD-9-CM* diagnosis codes used to identify acute infections

This supplemental material has been provided by the authors to give readers additional information about their work.

© 2021 Nam YH et al. *JAMA Network Open.*

eFigure 1. Observation time

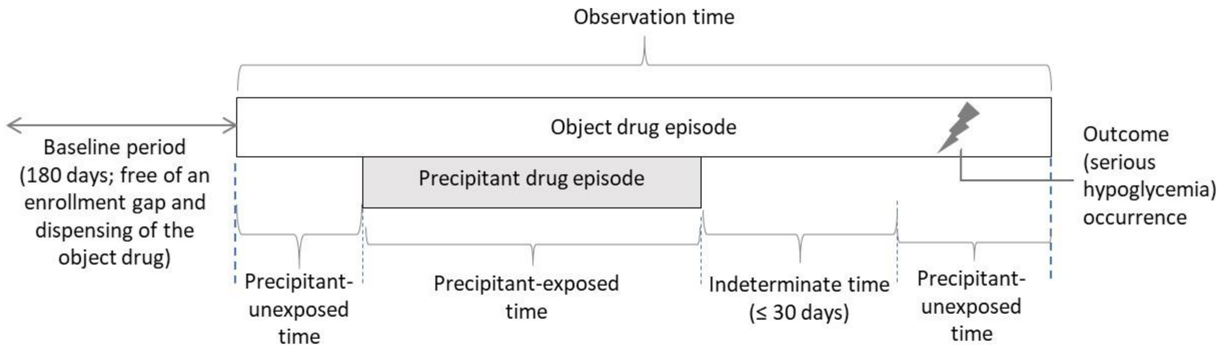

Figure S1. Observation time

Object drugs: insulin secretagogues or metformin. Precipitant drugs: calcium channel blockers. Observation time, defined as a period covered by object drug prescriptions, i.e., object drug episodes, was constructed for each object drug separately, and at least one outcome event occurrence during the observation time was required. Days' supply of an object drug was censored by dispensing of a different object drug, except when metformin (negative control object drug) was subsequently dispensed before the end of days' supply of an insulin secretagogue (object drug) for which we controlled for the indicator of metformin use as a time-varying variable in the statistical analysis without censoring the object drug's days supply. A drug episode was defined as a unit of consecutive prescriptions (a 7-day gap was allowed between contiguous prescriptions and at the end of the last prescription to account for potential incomplete adherence). An object drug episode began at the first dispensing date of that episode and ended by the end of days' supply of the episode (including a 7-day grace period), Medicaid disenrollment, or end of dataset, whichever occurred first. A precipitant drug episode was allowed to begin on, before, or after the start date of the object drug episode. More than one precipitant drug episode was allowed within an object drug's observation time. Indeterminate time lasted up to 30 days or until another prescription for the precipitant was dispensed. Observation time censored by death was included in a sensitivity analysis.

© 2021 Nam YH et al. *JAMA Network Open.*

eFigure 2. Flowchart of the identification of study samples

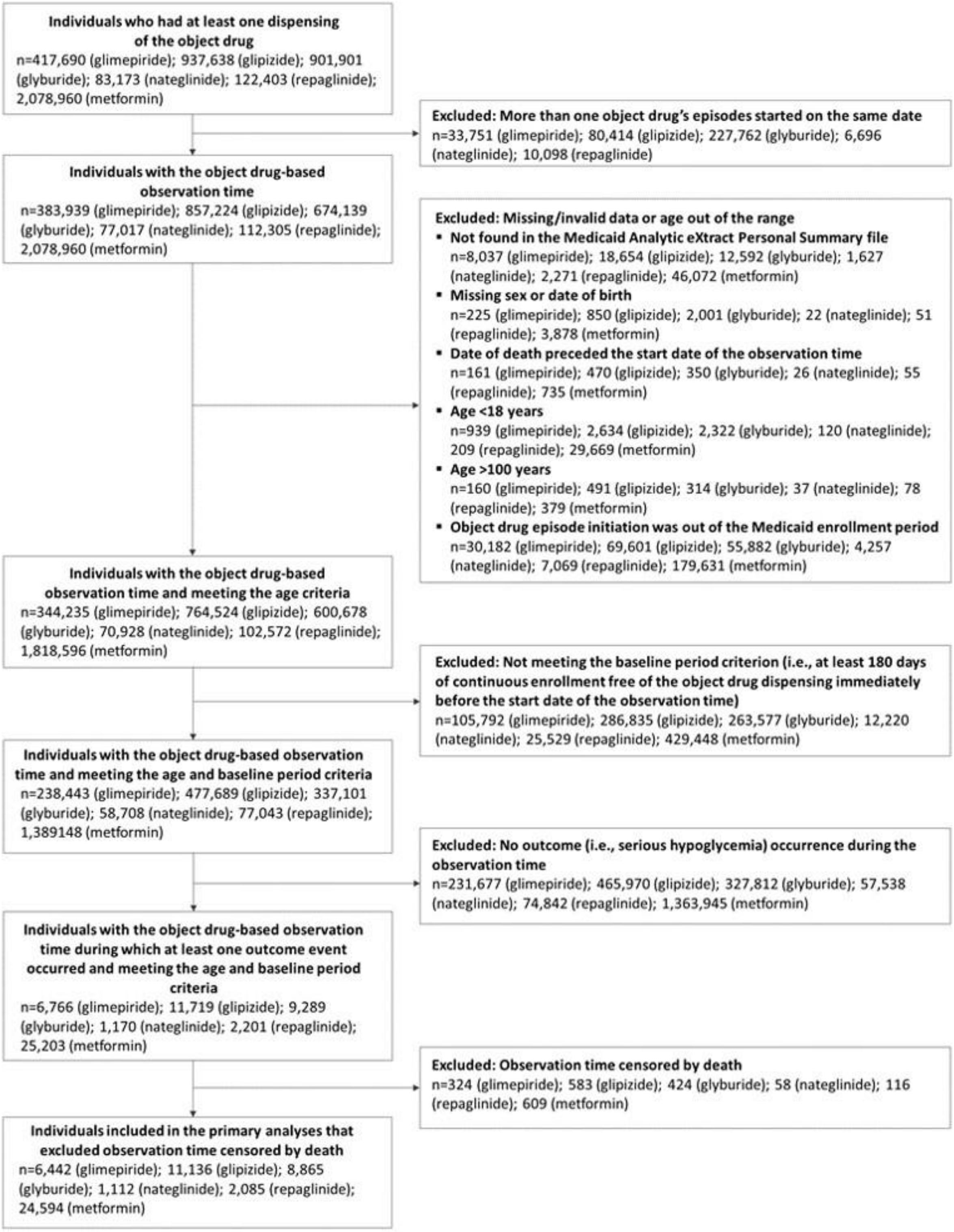

Figure S2. Flowchart of the Identification of study samples

Study samples were identified for each object drug separately. Object drugs (glimepiride, glipizide, glyburide, nateglinide, repaglinide, and metformin) are indicated in the parentheses.

© 2021 Nam YH et al. *JAMA Network Open.*

eTable 1. Prespecified time-varying potential confounders controlled for with conditional Poisson regression model

**Drugs that may be associated with hypoglycemia[a]**	
Anti-infectives[b]	alatrofloxacin, cinoxacin, ciprofloxacin, chloramphenicol, chloroquine, enoxacin, gatifloxacin, gemifloxacin, grepafloxacin, levofloxacin, lomefloxacin, moxifloxacin, nalidixic acid, norfloxacin, ofloxacin, pentamidine, quinine, sparfloxacin, sulfamethoxazole, trimethoprim, trovafloxacin
Angiotensin converting enzyme inhibitors[c]	benazepril, captopril, enalapril, fosinopril, lisinopril, moexipril, perindopril, quinapril, ramipril, trandolapril
Angiotensin II receptor blockers[c]	losartan, candesartan, valsartan, irbesartan, eprosartan, olmesartan, telmisartan, azilsartan
Beta blockers[c]	acebutolol, atenolol, betaxolol, bisoprolol, carteolol, labetalol, metoprolol, nadolol, nebivolol, penbutolol, pindolol, propranolol, sotalol, timolol
Salicylates[c]	aminosalicylic acid, aspirin, bismuth subsalicylate, choline salicylate, magnesium salicylate, magnesium salicylate tetrahydrate, phenyl salicylate, salicylic acid, salsalate, sodium salicylate, sodium thiosalicylate
Others[c]	haloperidol, quinidine, clofibrate, disopyramide
**Drugs that may be associated with hyperglycemia[a]**	
Atypical antipsychotics[c]	aripiprazole, clozapine, iloperidone, lurasidone, olanzapine, paliperidone, quetiapine, risperidone, ziprasidone
Calcineurin inhibitors[c]	cyclosporine, sirolimus, tacrolimus
Corticosteroids[c]	betamethasone, budesonide, cortisone, dexamethasone, fluodrocortisone, hydrocortisone, methylprednisolone, prednisolone, prednisone, triamcinolone
Protease inhibitors[c]	amprenavir, atazanavir, darunavir, fosamprenavir, indinavir, lopinavir, nelfinavir, ritonavir, saquinavir, tipranavir
Furosemide[c]	furosemide
Thiazide and thiazide-like diuretics[c]	bendroflumethiazide, benzthiazide, chlorothiazide, chlorthalidone, hydrochlorothiazide, hydroflumethiazide, indapamide, methyclothiazide, metolazone, polythiazide, trichlormethiazide
**Drugs that may interact with insulin secretagogues[a]**	
CYP2C9 inhibitors	amiodarone[c], capecitabine[c], cotrimoxazole[b], efavirenz[c], fenofibrate[c], fluconazole[b], isoniazid[c], lovastatin[c], metronidazole[b], miconazole[b], oxandrolone[c], paroxetine[c], probenecid[c], sulfamethoxazole[b], sulfinpyrazone[c], teniposide[c], tigecycline[c], voriconazole[b], zafirlukast[c]
CYP3A4 inhibitors	Azithromycin[b], clarithromycin[b], erythromycin[b], simvastatin[c], gemfibrozil[c]
Insulin[c]	insulin
Other non-insulin antidiabetes drugs[c]	acarbose, exenatide, linagliptin, pioglitazone, pramlintide, rosiglitazone, saxagliptin
**Major non-chronic condition that may be associated with hypoglycemia[d]**	
Acute infections[b]	Acute infections identified by discharge diagnosis codes appearing at any position in inpatient or outpatient claims
**Others[e]**	Age, nursing home residence status, Medicaid-Medicare dual-enrollment status, concomitant metformin use when the object drug was an insulin secretagogue

CYP: cytochrome P450 enzyme. [a]Identified by National Drug Codes, dispensing date, and days' supply. [b]Measured as a person-day level binary variable indicating being dispensed or diagnosed on the current day or any time during the 15 days prior to the current day. [c]Measured as a person-day level binary variable indicating being dispensed on the current day (refers to each day during the observation time as current) or any time during the 30 days prior to the current day. [d]Identified by International Classification of Diseases, 9th Revision, Clinical Modification diagnosis codes (listed in Table S2) and service date. [e]Measured on the current day; binary variables except for age (continuous variable).

© 2021 Nam YH et al. *JAMA Network Open.*

eTable 2. ICD-9-CM diagnosis codes used to identify acute infections

ICD-9-CM code	Description
001.*	Cholera
002.*	Typhoid and paratyphoid fevers
003.*	Other salmonella infections
004.*	Shigellosis
005.*	Other food poisoning (bacterial)
006.*, except 006.1	Amebiasis
007.*	Other protozoal intestinal diseases
008.*	Intestinal infections due to other organisms
009.*	Ill-defined intestinal infections
018.0*	Miliary tuberculosis
020.*	Plague
021.*	Tularemia
022.*	Anthrax
023.*	Brucellosis
024	Glanders
025	Melioidosis
026.*	Rat-bite fever
027.*	Other zoonotic bacterial diseases
030.*	Leprosy
031.*	Diseases due to other mycobacteria
032.*	Diphtheria
033.*	Whooping cough
034.*	Streptococcal sore throat and scarlet fever
035	Erysipelas
036.*	Meningococcal infection
037	Tetanus
038.*	Septicemia
039.*, except 039.4	Actinomycotic infections
040.*, except 040.1	Other bacterial diseases
041.*, except 041.81	Bacterial infection in conditions classified elsewhere and of unspecified site
045.*	Acute poliomyelitis
047.*	Meningitis due to enterovirus
048	Other enterovirus diseases of central nervous system
049.*	Other non-arthropod-borne viral diseases of central nervous system
050.*	Smallpox
051.*	Cowpox and paravaccinia
052.*	Chickenpox
053.*, except 053.12, 053.13	Herpes zoster
054.*	Herpes simplex
055.*	Measles
056.*	Rubella
057.*	Other viral exanthemata
058.*	Other human herpesvirus
059.*	Other poxvirus infections
060.*	Yellow fever
061	Dengue
062.*	Mosquito-borne viral encephalitis
063.*	Tick-borne viral encephalitis
064	Viral encephalitis transmitted by other and unspecified arthropods

© 2021 Nam YH et al. *JAMA Network Open.*

ICD-9-CM code	Description
065.*	Arthropod-borne hemorrhagic fever
066.*	Other arthropod-borne viral diseases
070.*, except 070.22, 070.23, 070.32, 070.33, 070.44, 070.54	Viral hepatitis
071	Rabies
072.*	Mumps
073.*	Ornithosis
074.*	Specific diseases due to coxsackie virus
075	Infectious mononucleosis
076.*	Trachoma
077.*	Other diseases of conjunctiva due to viruses and chlamydiae
078.*	Other diseases due to viruses and chlamydiae
079.*, except 079.51, 079.52, 079.53	Viral and chlamydial infection in conditions classified elsewhere and of unspecified site
080	Louse-borne (epidemic) typhus
081.*	Other typhus
082.*	Tick-borne rickettsioses
083.*	Other rickettsioses
084.*	Malaria
085.*	Leishmaniasis
086.*	Trypanosomiasis
087.*	Relapsing fever
088.*	Other arthropod-borne diseases
090.*	Congenital syphilis
091.*	Early syphilis symptomatic
092.*	Early syphilis latent
093.*	Cardiovascular syphilis
094.*	Neurosyphilis
095.*	Other forms of late syphilis with symptoms
096	Late syphilis, latent
097.*	Other and unspecified syphilis
098.*, except 098.2, 098.3*	Gonococcal infections
099.*	Other venereal diseases
100.*	Leptospirosis
101	Vincent's angina
103.*	Pinta
104.*	Other spirochetal infection
110.*	Dermatophytosis
111.*	Dermatomycosis other and unspecified
112.*	Candidiasis
114.*, except 114.4	Coccidioidomycosis
115.*	Histoplasmosis
116.*, except 116.2	Blastomycotic infection
118	Opportunistic mycoses
121.*	Other trematode infections
122.*	Echinococcosis
123.*	Other cestode infection
124	Trichinosis
125.*	Filarial infection and dracontiasis

© 2021 Nam YH et al. *JAMA Network Open.*

ICD-9-CM code	Description
126.*	Ancylostomiasis and necatoriasis
127.*	Other intestinal helminthiases
128.*	Other and unspecified helminthiases
129	Intestinal parasitism, unspecified
130.*	Toxoplasmosis
131.*	Trichomoniasis
132.*	Pediculosis and phthirus infestation
133.*	Acariasis
134.*	Other infestation
135	Sarcoidosis
136.*	Other and unspecified infectious and parasitic diseases
320.*	Bacterial meningitis
321.*	Meningitis due to other organisms
322.*	Meningitis of unspecified cause
323.*	Encephalitis myelitis and encephalomyelitis
460	Acute nasopharyngitis [common cold]
461.*	Acute sinusitis
462	Acute pharyngitis
463	Acute tonsillitis
464.*	Acute laryngitis and tracheitis
465.*	Acute upper respiratory infections of multiple or unspecified sites
466.*	Acute bronchitis and bronchiolitis
480.*	Viral pneumonia
481	Pneumococcal pneumonia [Streptococcus pneumoniae pneumonia]
482.*	Other bacterial pneumonia
483.*, except 483.0	Pneumonia due to other specified organism
484.*	Pneumonia in infectious diseases classified elsewhere
485	Bronchopneumonia, organism unspecified
486	Pneumonia, organism unspecified
487.*	Influenza
488.*	Influenza due to certain identified influenza viruses
590.1*	Acute pyelonephritis
595.0	Acute cystitis
598.0*	Urethral stricture due to infection
599.0	Urinary tract infection, site not specified

© 2021 Nam YH et al. *JAMA Netw Open*.
